# Compensatory Protection of Thioredoxin-Deficient Cells from Etoposide-Induced Cell Death by Selenoprotein W via Interaction with 14-3-3

**DOI:** 10.3390/ijms221910338

**Published:** 2021-09-25

**Authors:** Hyunwoo Kang, Yeong Ha Jeon, Minju Ham, Kwanyoung Ko, Ick Young Kim

**Affiliations:** Laboratory of Cellular and Molecular Biochemistry, Department of Life Sciences, Korea University, Seoul 02841, Korea; khw094@korea.ac.kr (H.K.); yeongha0820@cellabmed.com (Y.H.J.); minjham@hotmail.com (M.H.)

**Keywords:** selenoprotein W, thioredoxin, 14-3-3, Akt, cell death

## Abstract

Selenoprotein W (SELENOW) is a 9.6 kDa protein containing selenocysteine (Sec, U) in a conserved Cys-X-X-Sec (CXXU) motif. Previously, we reported that SELENOW regulates various cellular processes by interacting with 14-3-3β at the U of the CXXU motif. Thioredoxin (Trx) is a small protein that plays a key role in the cellular redox regulatory system. The CXXC motif of Trx is critical for redox regulation. Recently, an interaction between Trx1 and 14-3-3 has been predicted. However, the binding mechanism and its biological effects remain unknown. In this study, we found that Trx1 interacted with 14-3-3β at the Cys32 residue in the CXXC motif, and SELENOW and Trx1 were bound at Cys191 residue of 14-3-3β. In vitro binding assays showed that SELENOW and Trx1 competed for interaction with 14-3-3β. Compared to control cells, Trx1-deficient cells and SELENOW-deficient cells showed increased levels of both the subG1 population and poly (ADP-ribose) polymerase (PARP) cleavage by etoposide treatment. Moreover, Akt phosphorylation of Ser473 was reduced in Trx1-deficient cells and was recovered by overexpression of SELENOW. These results indicate that SELENOW can protect Trx1-deficient cells from etoposide-induced cell death through its interaction with 14-3-3β.

## 1. Introduction

Thioredoxin (Trx) is a ubiquitous, antioxidant protein containing a highly conserved Cys-X-X-Cys (CXXC) motif and plays an important role in the redox regulatory system [1]. Trx can reduce the oxidized Cys residues of target proteins, and the oxidized Trx is then reduced by thioredoxin reductase (TrxR) [2]. Trx interacts with various proteins involved in the regulation of cellular signaling pathways, including cell survival and proliferation. For example, Trx binds to phosphatase and tensin homolog (PTEN) and inhibits the lipid phosphatase activity of PTEN, which results in increased Akt activity in the cells [3]. Apoptosis signal-regulating kinase (ASK), which promotes apoptosis, is also a target protein of Trx. Ask1 activity is inhibited by Trx1 binding [4,5]. Moreover, Trx1 is known to be highly expressed in cancer cells [6,7].

Trx1 also plays an important role in cell survival [8,9,10,11]. Recently, it was suggested that Akt phosphorylation at Ser473 is upregulated by Trx1 [12,13]. Therefore, in Trx1-deficient cells, phosphorylation at Ser473 of Akt was found to be inhibited [14]. It has also been reported that phosphorylation of Akt at Ser473 can be regulated by the interaction between the 14-3-3 protein and Rictor, a component of the mechanistic target of rapamycin complex 2 (mTORC2) [15,16], and that the binding of 14-3-3β to Rictor is inhibited by the binding of 14-3-3β to selenoprotein W (SELENOW) [17].

SELENOW is the smallest selenoprotein that contains a conserved Cys-X-X-Sec (CXXU) motif, which corresponds to the CXXC redox motif of Trx1 [18]. The intramolecular selenylsulfide bond in the CXXU motif of SELENOW has been recently reported [19,20]. SELENOW is expressed in various tissues; however, it is highly abundant in the brain and muscles of mammals [21]. mRNA and protein expression of SELENOW is upregulated in the early stage of skeletal muscle cell differentiation [22]. Additionally, SELENOW is involved in the protection of cells from oxidative stress-induced cell death in a glutathione-dependent manner [23,24,25,26,27]. SELENOW interacts with the 14-3-3 protein and inhibits its interaction with other target proteins. For example, SELENOW reduced the binding of Rictor to 14-3-3β and increased the phosphorylation of Akt at Ser473 [17]. Downregulation of SELENOW induces cell cycle arrest in cancer cell lines, such as A549 and MCF7 cells, and makes the cells more sensitive to DNA damage by increasing the binding of 14-3-3β to Rictor or CDC25B [17,28]. Etoposide is a cancer chemotherapy drug which induces DNA damage in cells by inhibiting DNA religation activity of topoisomerase Ⅱ [29]. The subG1 population of DNA damaged cells by etoposide was increased in SELENOW-deficient cells compared to that in control cells [30].

The 14-3-3 protein plays an important role in diverse signaling processes [31]. The function of many cellular proteins is modulated by interactions with the 14-3-3 protein [32,33,34,35,36]. It exists as both homodimers and heterodimers in cells, and dimers can bind to phosphorylated Ser or Thr residues in target proteins [37,38]. However, it has also been reported that the 14-3-3 protein interacts with various target proteins in a phosphorylation-independent manner [39,40,41,42,43,44]. We previously reported that phosphorylation was not required for the interaction between SELENOW and 14-3-β, and Sec in the CXXU motif of SELENOW was identified as the binding site [30].

Similar to SELENOW, Trx1 is also predicted to bind to 14-3-3 [45,46]. Therefore, in this study, we investigated the interaction of Trx1 with 14-3-3β, the relationship between Trx1 and SELENOW, and the effect of these interactions on cell viability after DNA damage. 

## 2. Results

### 2.1. Cys32 in the CXXC Motif of Trx1 is Required for Interaction with 14-3-3β

**Figure 1:**
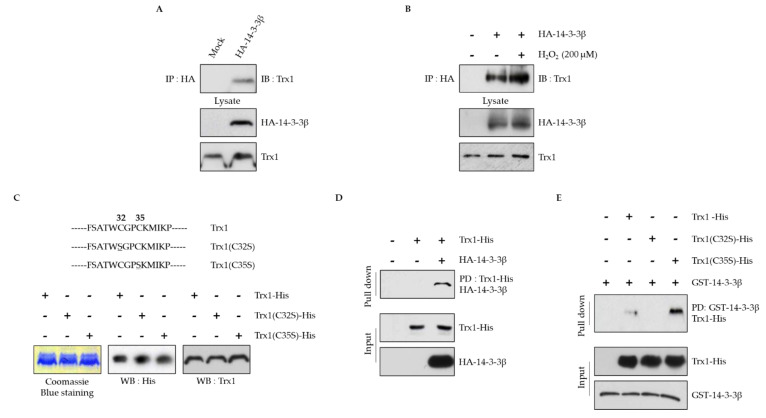
Trx1 interacts with 14-3-3β. (**A**) Human embryonic kidney 293 (HEK293) cells were transfected with HA-14-3-3β, and the cells were harvested 24 h after transfection. The cell extracts were immunoprecipitated with anti-HA antibody and then immunoblotted with anti-Trx1 antibody. (**B**) HEK293 cells were transfected with HA-14-3-3β for 24 h and then treated with H_2_O_2_ (200 μM) for 30 min. The cell extracts were immunoprecipitated with anti-HA antibody and then immunoblotted with anti-Trx1 antibody. (**C**) Sequences of mutant Trx1. The underlines and numbers indicate the mutation sites. Trx1-His, Trx1(C32S)-His, and Trx1(C35S)-His proteins were purified and verified by Coomassie staining (left) and immunoblotting with anti-His (middle) and anti-Trx1 (right) antibodies. (**D**) Purified Trx1-His protein was incubated with HEK293 cell lysates overexpressing HA-14-3-3β for 1 h. The mixtures were incubated with Ni-NTA beads for 1 h and then immunoblotted with anti-HA or anti-His antibodies. (**E**) Each purified Trx1-His, Trx1(C32S)-His, and Trx1(C35S)-His protein was incubated with purified GST-14-3-3β. The mixtures were incubated with glutathione beads for 1 h and then immunoblotted with anti-His or anti-GST antibodies. The data were obtained from three independent experiments.

The 14-3-3 protein regulates many signals by interacting with various proteins [32,33,34,35,36]. We previously reported that the interaction between 14-3-3β and SELENOW regulates sensitivity to DNA damage [30]. Sec of the CXXU motif is essential for the interaction. Similar to SELENOW, Trx1 has a conserved CXXC motif, which was also predicted to be a 14-3-3 binding partner [45,46]. To determine the interaction of Trx1 with 14-3-3β, we first performed an immunoprecipitation experiment using HEK293 cells transfected with HA-14-3-3β. Trx1 interacted with 14-3-3β, and this interaction was enhanced by H_2_O_2_ treatment (Figure 1A,B). To further confirm this interaction, a His-pull-down assay was performed. As Sec13 in the CXXU motif was required for the interaction of SELENOW with 14-3-3β [30], we constructed Trx1-His mutants in which Cys32 or Cys35 in the CXXC motif was changed to Ser. The mutants were designated Trx1(C32S)-His and Trx1(C35S)-His, respectively. The Trx1-His protein and Trx1 mutant proteins were expressed and extracted from *Escherichia coli* BL21(DE3) cells. The purified proteins were confirmed by Coomassie blue staining and Western blot assay (Figure 1C). The purified protein was used as bait in the His-pull-down assay against lysates of HEK293 cells overexpressing HA-14-3-3β. As shown in Figure 1D, Trx1-His interacted with HA-14-3-3β in vitro. Next, we investigated the binding sites of Trx1 for 14-3-3β. Using the purified proteins, a GST-pull-down assay was performed against the purified GST-14-3-3β protein. As shown in Figure 1E, Trx1-His and Trx1(C35S)-His interacted with purified GST-14-3-3β, but not Trx1(C32S)-His. These results indicate that similar to SELENOW, Trx1 also interacts with 14-3-3β and the Cys32 of the CXXC redox motif essential for this interaction.

### 2.2. The Cys191 Residue of 14-3-3β Is Identified as the Binding Site of SELENOW and Trx1

**Figure 2:**
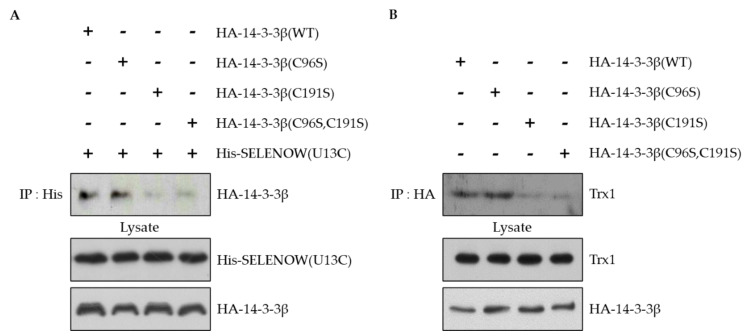
The Cys191 residue of 14-3-3β interacts with SELENOW and Trx1. (**A**) HEK293 cells were co-transfected with His-SELENOW(U13C) and HA-14-3-3β(WT, C96S, C191S or C96,191S). The cell extracts were immunoprecipitated with anti-His antibody, and immunoblotting was performed with anti-HA and anti-His antibodies. (**B**) HEK293 cells were co-transfected with HA-14-3-3β(WT, C96S, C191S or C96,191S). The cell extracts were immunoprecipitated with anti-HA antibody and immunoblotted with anti-Trx1 antibody or anti-HA antibody. The data were obtained from three independent experiments.

Sec13 in the CXXU motif of SELENOW is the binding site for 14-3-3β, and the interaction is redox-regulated [30]. In this study, we determined the binding site of 14-3-3β to SELENOW and Trx1. Human 14-3-3β has two Cys residues at positions 96 and 191 [47]. Mutants for 14-3-3β were constructed in which Cys96 or/and Cys191 were replaced by Ser. The mutants were designated as HA-14-3-3β(C96S), HA-14-3-3β(C191S), and HA-14-3-3β(C96, 191S). The interaction with SELENOW was then investigated via immunoprecipitation using HEK293 cells co-transfected with HA-14-3-3β and His-SELENOW(U13C). As shown in Figure 2A, both HA-14-3-3β(WT) and HA-14-3-3β(C96S) interacted with His-SELENOW(U13C). However, endogenous Trx1 did not interact with either HA-14-3-3β(C191S) or HA-14-3-3β(C96,191S) (Figure 2B). These results indicate that Cys191 of the 14-3-3β is required for interaction with both SELENOW and Trx1.

### 2.3. Interaction of 14-3-3β with Trx1 Is Regulated by SELENOW and Vice Versa

**Figure 3:**
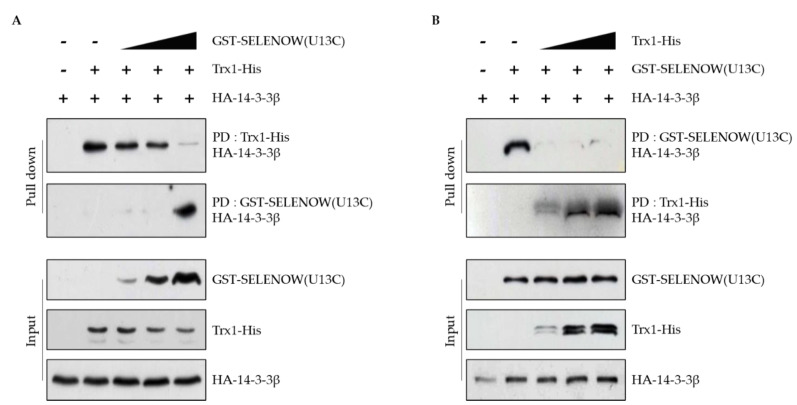
The interaction of Trx1 and SELENOW with 14-3-3β protein is competitive. (**A**) The mixture of purified Trx1-His protein (5 μg) and cell lysates overexpressing HA-14-3-3β (5 μg) was incubated with 0, 1, 2, or 5 μg GST-SELENOW(U13C) for 2 h. The mixtures were then incubated with glutathione or Ni-NTA beads for 1 h. The proteins were separated using non-reducing SDS-PAGE. The separated proteins were immunoblotted with anti-HA antibody. (**B**) The mixture of purified GST-SELENOW(U13C) protein (5 μg) and cell lysates overexpressing HA-14-3-3β (5 μg) was incubated with 0, 1, 2, or 5 μg Trx1-His for 2 h. The mixtures were incubated with glutathione or Ni-NTA beads for 1 h. The proteins were separated using non-reducing SDS-PAGE. The separated proteins were immunoblotted with anti-HA antibody.

Since both SELENOW and Trx1 interacted with the same residue of 14-3-3β, Cys191, we investigated the relationship between these two proteins and their interaction with 14-3-3β. A pull-down assay was performed with purified Trx1-His against the lysates of HEK293 cells overexpressing HA-14-3-3β in different concentrations of purified GST-SELENOW(U13C). As shown in Figure 3A, the binding of Trx1 to 14-3-3β protein decreased with increasing amounts of SELENOW(U13C). A pull-down assay with purified GST-SELENOW(U13C) against the lysates of HEK293 cells overexpressing HA-14-3-3β showed that the interaction between SELENOW and 14-3-3β was also decreased by increasing concentration of Trx1-His in the reaction mixtures (Figure 3B). These results demonstrated that Trx1 and SELENOW may compete to interact with the same site as on the 14-3-3β.

### 2.4. Deficiency of Trx1 and SELENOW Increases the Sensitivity of Cells to Etoposide-Induced Cell Death

**Figure 4:**
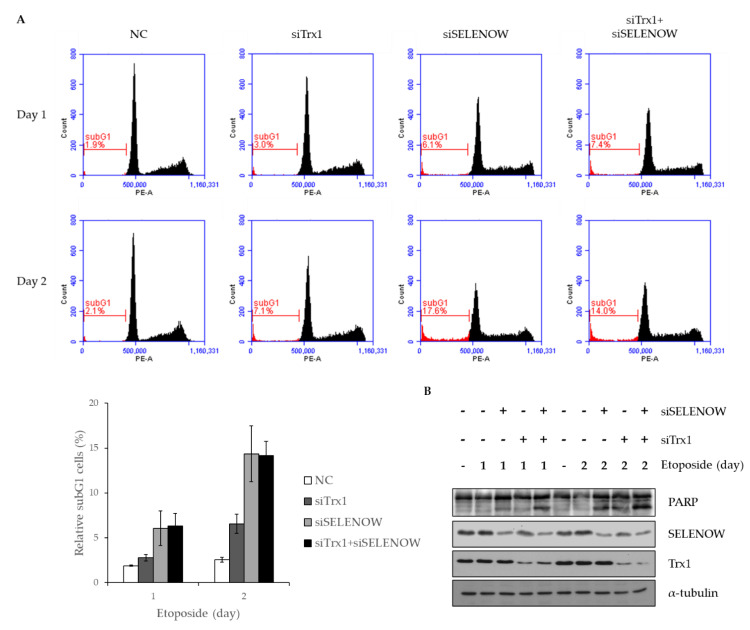
The cell death induced by DNA damage is regulated by the cooperation of SELENOW and Trx1. (**A**) Breast carcinoma MCF7 cells were transfected with siSELENOW or/and siTrx1 and incubated with etoposide (25 μM) for 24 or 48 h to induce DNA damage. Cells were harvested and subjected to FACS analysis (upper panel). The subG1 population is graphically represented (lower panel). The graph indicates the results from three independent experiments. The error bars in the graph indicate ±standard deviations (s.d.). (**B**) MCF7 cells were transiently transfected with siSELENOW or/and siTrx1. After 24 h, the cells were treated with etoposide (25 μM) for 24 or 48 h. The cells lysates were analyzed using Western blot assay using the indicated antibodies.

A previous report suggested that downregulation of Trx1 increased sensitivity to DNA damage by reducing cyclin D1 expression and phosphorylation of ERK1/2 in A549 and MCF7 cells [48]. It has been reported that the subG1 phase is increased in SELENOW-deficient MCF7 cells. SELENOW-deficient cells were also more sensitive to DNA damage induced by etoposide in A549, T47D, and MCF7 cells. This was observed due to the disruption in the binding of 14-3-3β to Rictor by SELENOW, which regulates phosphorylation of Akt at Ser 473 [17]. Therefore, we next examined the effect of double deficiency of Trx1 and SELENOW on cell survival under etoposide treatment conditions. MCF7 cells were transfected with siSELENOW and/or siTrx1 and then incubated with etoposide for 24 or 48 h to induce DNA damage. The cell cycle distribution was examined using FACS analysis. The subG1 phase population increased in SELENOW-deficient cells as shown in a previous report [17]. In this study, we found that the subG1 population also increased in etoposide treated-Trx1-deficient cells. The subG1 population of SELENOW-deficient cells was higher than that of Trx1-deficient cells (Figure 4A). We have previously shown that poly (ADP-ribose) polymerase (PARP) is rapidly cleaved in SELENOW-deficient cells compared to that in control cells under the DNA damage conditions induced by etoposide [28,30]. In this study, we found that PARP cleavage induced by etoposide also increased in Trx1-deficient cells, and it was further enhanced by double deficiency of SELENOW and Trx1 (Figure 4B). These results suggest that both Trx1 and SELENOW deficiency increase the cell death induced by DNA damage.

### 2.5. Decreased Akt Phosphorylation by Downregulation of Trx1 is Restored by SELENOW Overexpression

**Figure 5:**
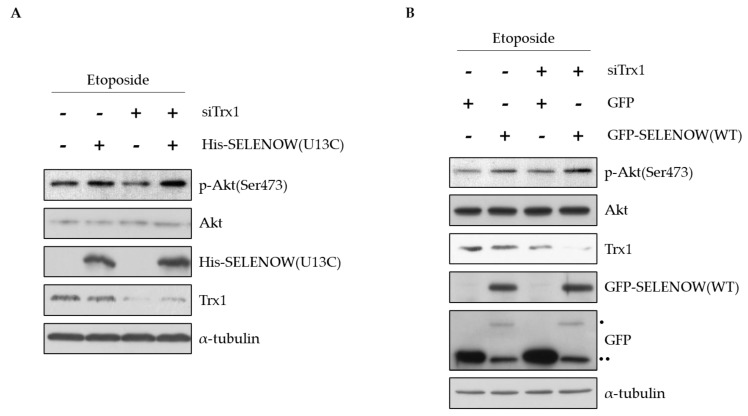
Decreased Akt phosphorylation of Trx1-deficent cells is restored by SELENOW. (**A**) MCF7 cells were transfected with siTrx1 or His-SELENOW(U13C), or co-transfected with siTrx1 and His-SELENOW(U13C) for 24 h and then treated with etoposide (25 μM) for 10 h. The cells lysates were analyzed by immunoblotting with the indicated antibodies. (**B**) MCF7 cells were co-transfected with siTrx1 and GFP-SELENOW(WT) for 24 h and further treated with etoposide for 10 h. The cells lysates were analyzed by immunoblotting with the indicated antibodies. • Full length GFP-SELENOW, •• pre-terminated GFP-SELENOW.

Akt activation promotes cell survival [49,50]. The binding of SELENOW to 14-3-3β reduced the interaction between Rictor and 14-3-3β, leading to Akt phosphorylation at Ser473 [51]. As the binding mechanism of Trx1 to 14-3-3β was similar to that of SELENOW, we investigated whether Akt phosphorylation affected by Trx1 depletion can be regulated by SELENOW overexpression. Previous reports showed that SELENOW-deficient cells or Trx1-deficient cells decreased phosphorylation of Akt, and the levels were recovered in SELENOW-deficient cells by ectopic expression of SELENOW(U13C) [14,17,30,51]. In this study, we found that downregulation of Akt phosphorylation by Trx1 depletion was recovered when SELENOW(U13C) or SELENOW(WT) was overexpressed in the cells (Figure 5A,B). These results suggest that SELENOW can compensate for the regulation of Akt phosphorylation by Trx1 via interaction with 14-3-3β in cells.

## 3. Discussion

The 14-3-3 protein interacts with various binding partners to play important roles in diverse signaling processes, including bacterial pathogenesis, cell growth, and development [52,53]. In particular, 14-3-3 functions as a regulator of cell survival under DNA damage [15]. mTORC2 is activated during DNA damage and it phosphorylates Akt at Ser473 [54]. Phosphorylated Akt provides a survival signal to cells from apoptotic stimuli [55]. Akt phosphorylation is inhibited by the interaction between Rictor and 14-3-3 [16]. Therefore, the regulation of the 14-3-3 protein is important for cell survival. 

Our previous works showed that the binding of SELENOW to 14-3-3β inhibits the binding of 14-3-3β to its target proteins and regulates various cellular processes, such as cell growth, muscle differentiation, cellular oxidative stress, and cell progression [17,28,56].

SELENOW is a protein containing a Trx-like fold and a CXXU motif, which corresponds to the CXXC motif of Trx [18]. Trx is a small redox protein involved in various processes, such as cell redox homeostasis, cell growth, DNA repair, and cell survival [57,58,59,60]. Trx1 binds to proteins such as CDC25, PTEN, and RNR, which are involved in various cellular processes [3,61,62]. Recently, it has been suggested that 14-3-3β might be a target for Trx-like proteins [45,46]. 

In this study, we found a direct interaction between Trx1 and 14-3-3β by immunoprecipitation and pull-down assays, and the binding was increased during oxidative stress (Figure 1). Trx1 contains two Cys residues, Cys32 and Cys35, in the CXXC motif which are used to reduce the target proteins [63]. Sec13 in the CXXU motif of SELENOW was identified as the binding site for 14-3-3β [30]. Therefore, we constructed Trx1 mutants in which Cys32 or Cys35 was replaced with Ser and Cys32 was identified as the binding site for the 14-3-3β (Figure 1C,E). 

Previously, we identified the binding site of SELENOW in the interaction between SELENOW and 14-3-3β, but not the binding site of 14-3-3β. In this study, we determined the binding site of 14-3-3β to SELENOW and investigated whether this binding site is also involved in the interaction with Trx1. Since the interaction of SELENOW with 14-3-3β was redox-regulated, we mutated two Cys residues, Cys96 and Cys191, in 14-3-3β to Ser residues. As shown in Figure 2A,B, the Cys191 of 14-3-3β was required for the interaction with Trx1 or SELENOW, indicating that 14-3-3β Cys191 is a common binding site for Trx1 and SELENOW. Previously, the Cys191 of 14-3-3β was also predicted to bind to SELENOW by computational calculations based on NMR data [47]. 

Since the binding of SELENOW and Trx1 to 14-3-3β occurs at the same site, Cys191, the relationship between SELENOW and Trx1 in the binding to 14-3-3β was investigated. The binding of Trx1 to 14-3-3β protein decreased with increasing amounts of SELENOW, and the binding of SELENOW to 14-3-3β also decreased with increasing amounts of Trx1 (Figure 3). These results suggest that the 14-3-3β signaling pathway can be cooperatively regulated by Trx1 and SELENOW. There is no interaction between Trx1 and SELENOW [27]. 

In this study, we found that Trx1 interacted with 14-3-3β. Since sensitivity to DNA damage was increased in SELENOW-deficient cells by etoposide treatment [30], we also determined the effect of Trx1-deficiency on cell viability. Compared to control cells, the subG1 population was increased in SELENOW-deficient cells or Trx1-deficient cells treated with etoposide. Furthermore, PARP was rapidly cleaved in SELENOW-deficient and Trx1-deficient cells compared with that in control cells after treatment with etoposide (Figure 4). This suggests that cell death induced by DNA damage is regulated by both SELENOW and Trx1.

SELENOW has been reported to control cell survival by interacting with 14-3-3β [30]. SELENOW activates the mTORC2/Akt pathway for Akt phosphorylation at Ser473 by interrupting the binding of Rictor to 14-3-3β. In SELENOW-deficient cells, the binding of 14-3-3β to Rictor is enhanced, resulting in the inactivation of Akt by inhibiting phosphorylation at Ser473 [16,17]. Akt inactivation decreases the viability of cells upon treatment with etoposide.

Therefore, we next investigated whether Akt phosphorylation was also regulated by the interaction of Trx1 with 14-3-3β. Phosphorylation of Akt at Ser473 was decreased by depletion of Trx1 in etoposide-treated MCF7 cells and was restored by the expression of SELENOW(U13C) (Figure 5A). Using the wild-type SELENOW containing Sec in the CXXU motif, we confirmed the compensation of SELENOW for Trx1 deficiency (Figure 5B). 

Taken together, these results suggest that Trx1 regulates cell survival through interaction with 14-3-3β and that SELENOW compensates Trx1-deficient cells against cell death induced by etoposide treatment.

## 4. Materials and Methods

### 4.1. Cell Culture, Transfection, and Reagents

Breast carcinoma MCF7 cells were grown in RPMI 1640 medium containing 10% fetal bovine serum at 37 °C in 5% CO_2_. Human embryonic kidney 293 (HEK 293) cells were grown in DMEM containing 10 % FBS at 37 °C in 5% CO_2_. The cells were seeded at a density of 5 × 10^5^ cells in 60 mm dishes for transient transfection. Twelve hours after seeding, the cells were transfected using ScreenFect A plus (ScreenFect GmbH, Eggenstein-Leopoldshafen, Germany) and Lipofectamine 2000 (Invitrogen, Carlsbad, CA, USA), according to the manufacturer’s instructions. To measure the sensitivity to etoposide, MCF7 cells were treated with 25 μM etoposide (Sigma, St. Louis, MO, USA) after transfection and harvested.

### 4.2. RNA Interference and Plasmids

The siSELENOW used in this study was designed by Invitrogen. The sequence for human siSELENOW was as follows: siSELENOW 5′-CCA CCG GGU UCU UUG AAG UGA UGG U-3′. Human siTrx1 was purchased from Qiagen (Valencia, CA, USA). GFP-SELENOW(WT) was generated using the pEGFP-C2 plasmid from wild-type mouse SELENOW (Origene, Rockville, MD, USA). His-SELENOW(U13C) and HA-14-3-3β plasmids have been described previously [28,30].

### 4.3. Western Blot Assay

Cells were harvested and lysed as previously described [30]. Whole cell lysates were separated using SDS-PAGE and transferred to a PVDF membrane. Membrane was blocked with 5% skim milk for 1h and incubated with specific antibodies at 4 °C overnight with rotation. The antibodies were obtained from the following sources: anti-poly (ADP-ribose) polymerase (PARP) and anti-Akt, anti-phospho-Akt (Ser 473) were from Cell Signaling (Danvers, MA, USA); anti-GST and anti-GFP antibodies were from Santa Cruz Biotechnology (Santa Cruz, CA, USA); anti-His and anti-HA were from ABM (Richmond, BC, Canada); anti-α-tubulin and anti-Trx1 were from AB Frontier (Seoul, Republic of Korea); anti-SELENOW was from Origene (Rockville, MD, USA). After incubating with HRP conjugated secondary antibody for 1 h, immunoreactive bands were visualized using a West Pico Enhanced ECL Detection kit (Pierce, Rockford, IL, USA).

### 4.4. Immunoprecipitation

Cells were lysed with immunoprecipitation buffer as previously described [30]. The lysates were mixed with antibodies overnight at 4 °C with rotation. Immune complexes were incubated with Protein A or G beads for 1.5 h at 4 °C with rotation. The lysates were washed two times and boiled with SDS sample buffer for 3 min. The samples were separated and detected as described above.

### 4.5. Protein Purification

The mouse GST-SELENOW protein, GST-14-3-3β and human Trx1-His mutants were expressed and purified in *E. coli*. Briefly, BL21 (DE3) competent cells were transformed with mouse GST-SELENOW and human Trx1-His mutants in pGEX 4T-1 (Amersham Biosciences, Chalfont, UK) and pET26B (Novagen, Madison, WI, USA) plasmids. The proteins were induced by 1 mM IPTG for 16 h at 18 °C and purified using glutathione and Ni-NTA beads as described previously [27,30].

### 4.6. Pull-Down Assay

Purified proteins were incubated with cell lysate or recombinant proteins in pull-down buffer containing 20 mM HEPES (pH 7.5), 1 mM EDTA and 1 mM for 2 h at 4 °C with rotation, followed by glutathione and NI-NTA beads for 1 h. The beads were washed three times with the wash buffer and then eluted [27,30].

### 4.7. Flow Cytometry

MCF7 cells were fixed overnight in ice-cold 70% ethanol. Cells were collected by centrifugation and resuspended in PBS. The resuspended cells were treated with RNase A (100 μg/mL) for 30 min at RT, and the cells were then stained with propidium iodide (10 μg/mL). Subsequently, cell cycle distribution was analyzed using a FACS Accuri flow cytometer (BD Bioscience, San Jose, CA, USA).

## Data Availability

The data presented in this study are available upon request from the corresponding author.
